# Investigation of a Novel Salt Stress-Responsive Pathway Mediated by Arabidopsis DEAD-Box RNA Helicase Gene *AtRH17* Using RNA-Seq Analysis

**DOI:** 10.3390/ijms21051595

**Published:** 2020-02-26

**Authors:** Hye-Yeon Seok, Linh Vu Nguyen, Doai Van Nguyen, Sun-Young Lee, Yong-Hwan Moon

**Affiliations:** 1 Institute of Systems Biology, Pusan National University, Busan 46241, Korea; seokhyeon@pusan.ac.kr; 2 Department of Integrated Biological Science, Pusan National University, Busan 46241, Koreadoai.vn@gmail.com (D.V.N.); 3 Biological Systems and Engineering Division, Lawrence Berkeley National Laboratory, Berkeley, CA 94720, USA; symoonlee@lbl.gov; 4 Department of Molecular Biology, Pusan National University, Busan 46241, Korea

**Keywords:** Arabidopsis, AtRH17, DEAD-box RNA helicase, RNA-Sequencing, salt stress, stress-responsive pathway

## Abstract

Previously, we reported that overexpression of *AtRH17*, an Arabidopsis DEAD-box RNA helicase gene, confers salt stress-tolerance via a pathway other than the well-known salt stress-responsive pathways. To decipher the salt stress-responsive pathway in *AtRH17*-overexpressing transgenic plants (OXs), we performed RNA-Sequencing and identified 397 differentially expressed genes between wild type (WT) and *AtRH17* OXs. Among them, 286 genes were upregulated and 111 genes were downregulated in *AtRH17* OXs relative to WT. Gene ontology annotation enrichment and KEGG pathway analysis showed that the 397 upregulated and downregulated genes are involved in various biological functions including secretion, signaling, detoxification, metabolic pathways, catabolic pathways, and biosynthesis of secondary metabolites as well as in stress responses. Genevestigator analysis of the upregulated genes showed that nine genes, namely, *LEA4-5*, *GSTF6*, *DIN2*/*BGLU30*, *TSPO*, *GSTF7*, *LEA18*, *HAI1*, *ABR*, and *LTI30*, were upregulated in Arabidopsis under salt, osmotic, and drought stress conditions. In particular, the expression levels of *LEA4-5*, *TSPO*, and *ABR* were higher in *AtRH17* OXs than in WT under salt stress condition. Taken together, our results suggest that a high *AtRH17* expression confers salt stress-tolerance through a novel salt stress-responsive pathway involving nine genes, other than the well-known ABA-dependent and ABA-independent pathways.

## 1. Introduction

RNA helicases (RHs) are present in most prokaryotic and eukaryotic organisms, which catalyze the unwinding of DNA or the secondary structure of RNA, and thus, play essential roles in almost every aspect of genetic processes such as replication, transcription, translation, repair, and recombination [1,2]. RHs have been classified into six superfamilies (SF1–SF6) based on specific motif sequences and domain structures. Superfamily II (SF2), the largest helicase family, mainly consists of the DEAD-box RHs, which are named after the strictly conserved sequence “Asp-Glu-Ala-Asp” (D-E-A-D) [1,2]. Fifty-eight DEAD-box RHs have been identified in Arabidopsis [1,3].

DEAD-box RH has nine well-defined motifs (Q, I, Ia, Ib, and II–VI) divided into two helicase domains: domain 1 and domain 2 [1,3,4,5,6]. Each motif has been proposed to have specific helicase functions. The newly identified motif Q of DEAD-box RHs is supposed to control hydrolysis and ATP binding; motif I (popularly known as the Walker A motif) is associated with the interaction between ATP and Mg^2+^; motif Ia forms a groove binding to single-stranded DNA/RNA; motif II (also known as the Walker B motif) interacts with Mg^2+^; motif III is responsible for helicase and NTPase activities to perform RNA unwinding, and motif VI is a part of the ATP-binding cleft that is related to helicase and NTPase activities. The molecular functions of the remaining motifs (Ib, IV, and V) are still unclear [2,3]. In addition to these conserved motifs, the N-terminal and C-terminal extended regions have also been found in each DEAD-box RH protein, which vary greatly in terms of their size and composition; it has been proposed that they function in determining the substrate-binding specificities as subcellular localization signals or possibly interact with accessory components [7,8,9].

Recent studies have indicated the important roles of DEAD-box RHs in RNA biogenesis, pre-mRNA splicing, RNA export and storage, transcription, translation, and RNA decay as well as in organelle-specific RNA metabolism [2,3]. Furthermore, multiple studies have suggested that DEAD-box RHs also play essential roles in abiotic stress responses in plants through their functions in specific RNA processing events [10,11]. *AtRH7* participates in rRNA biogenesis and is involved in cold-tolerance in Arabidopsis [12]. *AtRH38*/*LOS4* has important roles in mRNA export and in cold and heat stress responses in Arabidopsis [13]. *AtRH47*/*RCF1* is also involved in cold stress-tolerance via proper splicing of pre-mRNAs of cold stress-responsive genes [14]. *STRS1* and *STRS2* reduce the expression of stress-responsive genes through RdDM-mediated gene silencing and function as negative regulators of the response to multiple abiotic stresses in Arabidopsis [15,16]. Despite these studies, the roles of DEAD-box RHs in abiotic stress response and/or abiotic stress-responsive pathways mediated by DEAD-box RHs are not well known, as yet.

Previously, we identified an Arabidopsis DEAD-box RH gene, *AtRH17*, using the activation tagging system. The activation tagging line, in which *AtRH17* was activated and *AtRH17*-overexpressing transgenic plants (OXs) showed the salt-tolerant phenotype at the seedling and mature plant stages [17]. However, transcript levels of the well-known salt stress-responsive genes such as *RD29A*, *RAB18*, *RD29B*, *RD22*, *COR47*, *DREB2A*, and *DREB2B*, were not higher in *AtRH17* OXs than in the wild type (WT) under salt stress conditions, suggesting that *AtRH17* is involved in salt stress-tolerance via a pathway other than the well-known salt stress-responsive pathway.

In the present study, to elucidate the salt stress-responsive pathway in *AtRH17* OXs, we performed RNA-Sequencing (RNA-Seq) and analyzed the expression of Arabidopsis genes in WT and *AtRH17* OXs. RNA-Seq analysis showed 286 upregulated and 111 downregulated genes in *AtRH17* OXs compared to WT. Gene Ontology (GO) annotation enrichment and Kyoto Encyclopedia of Genes and Genomes (KEGG) pathway analysis showed that the upregulated and downregulated genes are involved in various biological functions including stress responses. Further analysis of the upregulated genes suggested that overexpression of *AtRH17* confers salt stress-tolerance via pathway(s) involving *LEA4-5*, *GSTF6*, *DIN2*/*BGLU30*, *TSPO*, *GSTF7*, *LEA18*, *HAI1*, *ABR*, and *LTI30*, other than the well-known ABA-dependent and ABA-independent stress-responsive pathways in Arabidopsis.

## 2. Results

### 2.1. Transcriptomic Profiling of AtRH17 OXs

In a previous study, we reported that overexpression of *AtRH17* confers salt stress-tolerance at the seedling and mature plant stages. Interestingly, the well-known ABA-dependent and ABA-independent salt stress-responsive genes such as *RD29A*, *RAB18*, *RD29B*, *RD22*, *COR47*, *DREB2A*, and *DREB2B* showed similar or lower expression levels in *AtRH17* OXs than in WT under salt stress conditions [17], implying that salt stress-tolerance of *AtRH17* OXs is mediated by an uncharacterized pathway or mechanism other than the well-known stress-responsive pathways. In this study, to clarify the regulatory mechanism of salt stress-tolerance of *AtRH17* OXs, RNA-Seq was performed using 10-day-old WT and *AtRH17* OX whole seedlings. The mapping of RNA-Seq reads to the Col-0 genome was successful, with a mapping rate of 97.47% to 98.01% (Table 1). The number of mapped reads ranged from 26.4 to 30.3 million (Table 1). Genes having very low abundance were removed from the analysis, leaving 22,884 genes for further analysis.

### 2.2. Analysis of Up- and Downregulated Genes in AtRH17 OXs

**Figure 1:**
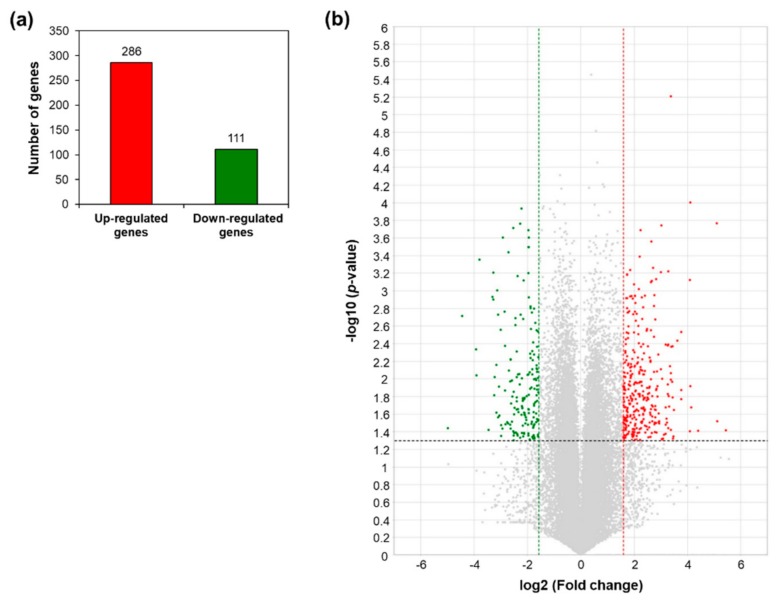
Differentially expressed genes in *AtRH17*-overexpressing transgenic plants (OXs) compared to wild type (WT) from the RNA-Sequencing (RNA-Seq) analysis. (**a**) Numbers of genes more than 3-fold up- and downregulated in *AtRH17* OXs compared to WT. (**b**) Volcano plot of differentially expressed genes (DEGs) identified between WT and *AtRH17* OXs (*p*-value < 0.05 and log2 ratio ≥ 1). The upregulated genes are represented by a red dot and downregulated genes by a green dot, grey dots indicate genes that are not differentially expressed.

A total of 397 differentially expressed genes (DEGs) between WT and *AtRH17* OX plants were identified with >3-fold differences in expression and a *p*-value < 0.05. Among these, 286 genes were upregulated and 111 genes were downregulated in *AtRH17* OXs (Figure 1a). The distribution of up- and downregulated genes is shown using volcano plot (Figure 1b).

The analysis of GO terms with three categories, namely, biological processes, molecular functions, and cellular components, using the selected genes is useful in predicting the altered biological and molecular processes. Therefore, 397 DEGs were subjected to GO enrichment analysis to determine their functional significance in *AtRH17* OXs. The upregulated genes were enriched in response to toxic substance, toxin catabolic process, glutathione metabolic process, and oxidation–reduction process in the biological process categories of GO annotation; glutathione transferase activity, lipid binding, heme binding, flavin adenine dinucleotide binding, and electron carrier activity in the molecular function categories; and extracellular region, apoplast, extracellular space, RNA polymerase II transcription factor complex, and cell wall in the cellular component categories (Supplementary Figure S1). The downregulated genes were enriched for plant-type cell wall organization, hydrogen peroxide catabolic process, and response to brassinosteroid in the biological process categories of GO annotation; structural constituent of cell wall, transcription factor activity, peroxidase activity in the molecular function categories; and cell wall, extracellular region, and plant-type cell wall in the cellular component categories (Supplementary Figure S2). GO enrichment analysis showed that many genes, which are involved in various biological and molecular processes such as signaling, secretion, detoxification, transcription, and stress responses, were up- and/or downregulated in *AtRH17* OXs.

We further analyzed the biological process categories of GO annotation to decipher the biological functions of the DEGs in *AtRH17* OXs. The upregulated genes were highly enriched in the biological process of oxidation–reduction (34 genes), defense response to fungus (14 genes), defense response (14 genes), response to oxidative stress (12 genes), response to salt stress (11 genes), response to toxic substance (10 genes), and response to water deprivation (10 genes) (Table 2), demonstrating that *AtRH17* might be involved in abiotic and biotic stress responses. In addition, *AtRH17* might be involved in the metabolic processes of sugar compounds and homeostasis (Table 2). The GO analysis of the downregulated genes revealed that the biological processes in which the downregulated genes are involved were mostly associated with oxidation–reduction (11 genes), defense response (seven genes), plant-type cell wall organization (six genes), response to oxidative stress (five genes), and hydrogen peroxide catabolic process (four genes) (Table 3), indicating that *AtRH17* might negatively regulate oxidative stress-related genes. Moreover, *AtRH17* can have a minor role in negative regulation of plant growth-related genes based on the enriched GO terms such as response to brassinosteroid, response to gibberellin, unidimensional cell growth, and response to ethylene (Table 3).

**Figure 2:**
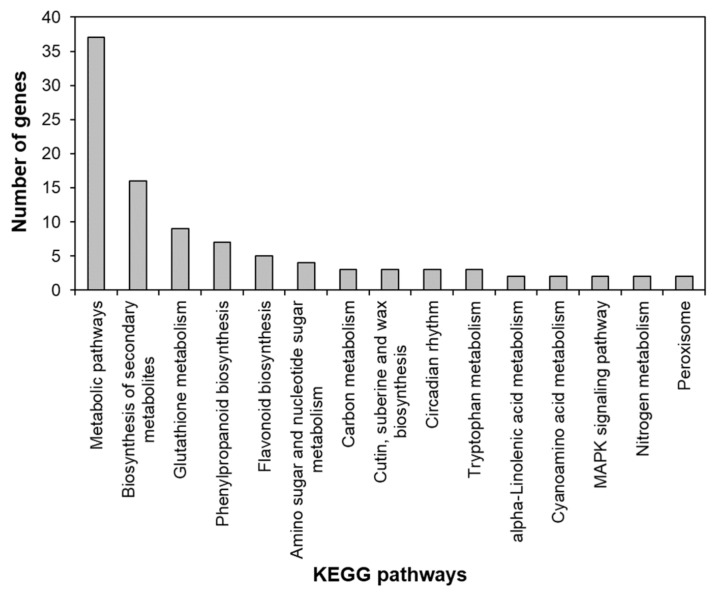
Kyoto Encyclopedia of Genes and Genomes (KEGG) pathway of upregulated genes in *AtRH17* OXs. KEGG pathway was analyzed using KEGG Mapper.

**Figure 3:**
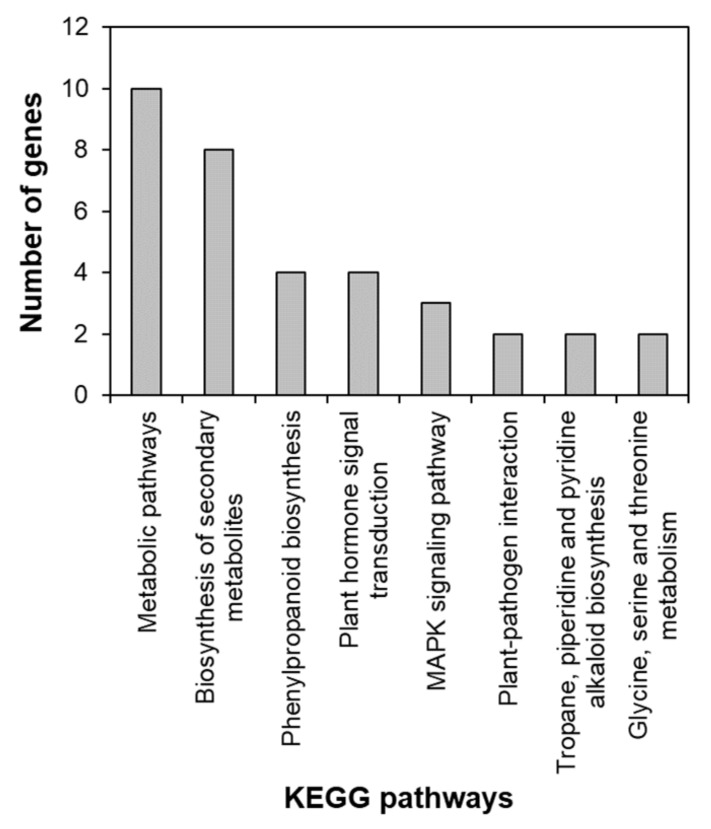
KEGG pathway of downregulated genes in *AtRH17* OXs. KEGG pathway was analyzed using KEGG Mapper.

**Figure 4:**
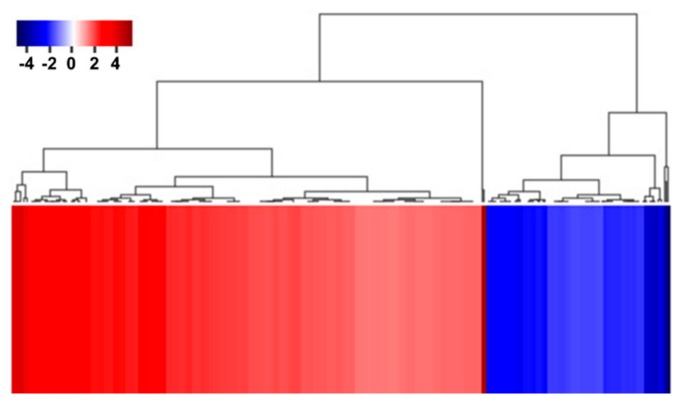
Hierarchical clustering of up- and downregulated genes in *AtRH17* OXs. Hierarchical clustering of up- and downregulated genes were performed using MultiExperiment Viewer (MeV).

To identify the functional pathways in which *AtRH17* is involved, KEGG pathway analysis was performed. Both the up- and downregulated genes were involved in metabolic pathways, biosynthesis of secondary metabolites, and phenylpropanoid biosynthesis (Figure 2 and Figure 3). In contrast, only the upregulated genes were involved in glutathione metabolism and flavonoid biosynthesis, whereas the downregulated genes were involved in plant hormone signal transduction and MAPK signaling pathway (Figure 2 and Figure 3), indicating that *AtRH17* might be involved in several functional pathways through the up- and/or downregulation of various related genes. We also performed hierarchical clustering to classify and identify the relationship among DEGs. The up- and downregulated genes were classified into individual hierarchies. The upregulated genes were classified into six groups, whereas the downregulated genes were classified into four groups (Figure 4).

**Figure 5:**
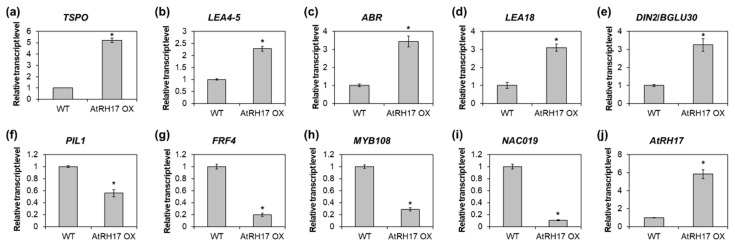
Verification of up- and downregulated genes in *AtRH17* OXs. Quantitative RT-PCR analysis of *TSPO* (**a**), *LEA4-5* (**b**), *ABR* (**c**), *LEA18* (**d**), *DIN2*/*BGLU30* (**e**), *PIL1* (**f**), *FRF4* (**g**), *MYB108* (**h**), *NAC019* (**i**), and *AtRH17* (**j**) in 10-day-old WT and *AtRH17* OX seedlings. *GAPc* was used as an internal control. Transcript levels in WT were set as 1. Three independent reactions were performed for each technical replicate. Two technical replicates were performed for each biological replicate. At least two biological replicates showed similar results, with one shown here. Error bars represent standard deviation (*n* = 6 reactions) and ^*^ indicates *t*-test *p* < 0.05.

To validate the RNA-Seq results by quantitative RT-PCR, we selected five upregulated genes, namely, *TSPO*, *LEA4-5*, *ABR*, *LEA18*, and *DIN2*/*BGLU30* and four downregulated genes, namely, *PIL1*, *FRF4*, *MYB108*, and *NAC019*. Thereafter, we analyzed the expression of the selected genes in 10-day-old WT and *AtRH17* OX seedlings. The expression of all five upregulated genes was significantly higher in *AtRH17* OXs than in WT (Figure 5a–e). Especially, *TSPO* expression was more than 5-fold higher in *AtRH17* OXs than in WT, whereas *LEA4-5* expression was 2.3-fold higher in *AtRH17* OXs than in WT (Figure 5a,b). The expression levels of *ABR*, *LEA18*, and *DIN2*/*BGLU30* were more than 3-fold higher in *AtRH17* OXs than in WT (Figure 5c–e). In contrast, the four downregulated genes showed lower expression in *AtRH17* OXs than in WT (Figure 5f–i). *PIL1* expression in *AtRH17* OXs was only almost half of that in WT (Figure 5f). *FRF4* and *MYB108* expression in *AtRH17* OXs were only one-fifth of that in WT (Figure 5g,h). Moreover, *NAC019* expression in *AtRH17* OXs was one-tenth of that in WT (Figure 5i). These results are consistent with the RNA-Seq results.

### 2.3. Identification of the Salt Stress-Tolerance Pathway in AtRH17 OXs

**Figure 6:**
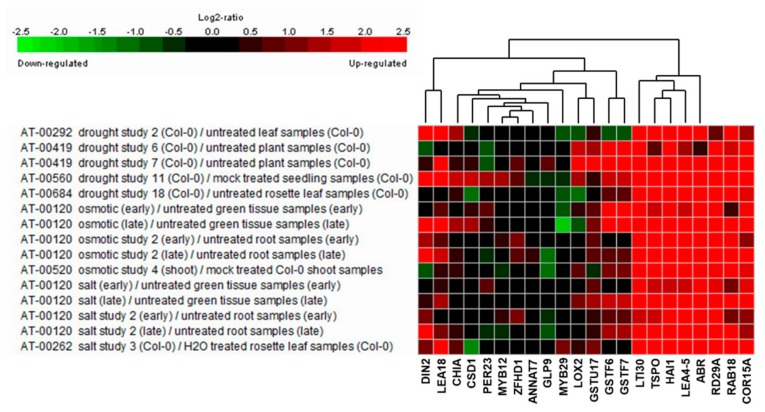
Expression analysis of 19 stress-responsive genes under drought, osmotic, and salt stress conditions using Genevestigator. *RD29A*, *RAB18*, and *COR15A* were used as marker genes for stress conditions.

To elucidate the salt stress-tolerance mechanism in *AtRH17* OXs, we isolated 19 upregulated genes enriched in the salt stress-related GO terms such as “response to salt” (GO:000965) and “response to water deprivation” (GO:0009414) (Table 4), indicating that these genes might function in salt stress-tolerance in *AtRH17* OXs. To see the expression patterns of the 19 genes under stress conditions, we performed expression analysis using Genevestigator under drought, osmotic, and salt stress conditions. Five experiments were used for each stress condition. We divided the 19 genes into three groups depending on their expression patterns. In the first group, eight genes, namely, *CHIA*, *CSD1*, *PER23*, *MYB12*, *ZFHD1*, *ANNAT7*, *GLP9*, and *MYB29*, did not respond to any of the stresses (Figure 6). In the second group, *LOX2* and *GSTU17* showed high expression levels under drought condition, whereas their levels did not increase under osmotic and salt stress conditions (Figure 6). In the third group, nine genes, namely, *DIN2*/*BGLU30*, *LEA18*, *GSTF6*, *GSTF7*, *LTI30*, *TSPO*, *HAI1*, *LEA4-5*, and *ABR*, showed high expression levels under all three examined stress conditions (Figure 6), demonstrating that they might be involved in salt stress-tolerance in *AtRH17* OXs (Table 5).

**Figure 7:**
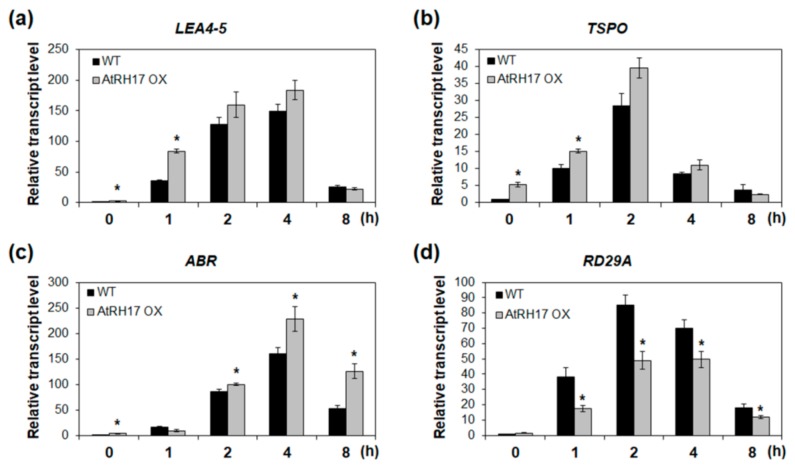
Expression analysis of three stress-responsive genes in *AtRH17* OXs under salt stress conditions. Quantitative RT-PCR analysis of *LEA4-5* (**a**), *TSPO* (**b**), *ABR* (**c**), and *RD29A* (**d**) in WT and *AtRH17* OX seedlings under 150 mM NaCl treatment for 0, 1, 2, 4, and 8 h. *GAPc* was used as an internal control. Transcript levels at 0 h in WT were set as 1. Three independent reactions were performed for each technical replicate. Two technical replicates were performed for each biological replicate. At least two biological replicates showed similar results, with one shown here. Error bars represent standard deviation (*n* = 6 reactions) and ^*^ indicates t-test * *p* < 0.05.

Next, we analyzed the expression of genes selected based on the Genevestigator analysis in WT and *AtRH17* OXs under salt stress condition to verify whether these genes are involved in the salt stress response of *AtRH17* OXs. *LEA4-5* and *ABR*, late embryogenesis abundant (LEA) protein genes, and *TSPO* were selected for expression analysis among the above nine genes. The biological functions of LEA proteins in osmotic stress-tolerance are well known [18,19]. TSPOs have also been shown to be responsive to salt and osmotic stresses and ABA [20,21]. Quantitative RT-PCR analysis showed that the expression levels of *LEA4-5*, *ABR*, and *TSPO* were higher in *AtRH17* OXs than in WT under salt stress conditions (Figure 7). Interestingly, the differences in expression levels of *LEA4-5* and *TSPO* between WT and *AtRH17* OXs were the highest under early salt stress conditions such as after 1 and 2 h of NaCl-treatment. Subsequently, the difference decreased gradually, and no difference was observed at 8 h after the NaCl-treatment (Figure 7a,b). However, the expression of *ABR* was almost similar in WT and *AtRH17* OXs until 2 h after the NaCl-treatment. Thereafter, the expression in *AtRH17* OXs significantly increased to more than that in WT until 8 h after the NaCl-treatment (Figure 7c), indicating that *LEA4-5* and *TSPO* might function in the early salt stress response, whereas *ABR* might be involved in the late salt stress response.

**Figure 8:**
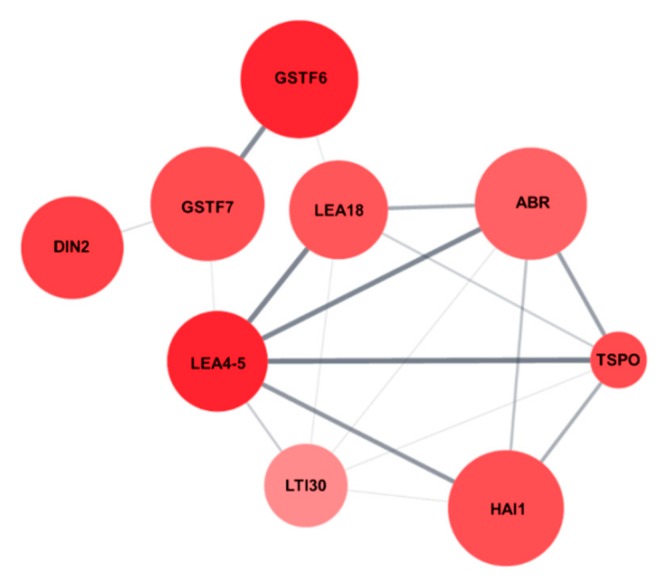
Protein network of the nine salt stress-responsive genes. Protein network interaction of the nine salt stress-responsive genes were analyzed using String Apps of Cytoscape.

Protein network analysis of the nine salt stress-responsive genes using Cytoscape String Apps revealed that the nine genes were tightly connected and interacted with each other (Figure 8), indicating that the salt stress-tolerance in *AtRH17* OXs might be the result of precise upregulation of these nine genes.

## 3. Discussion

In this study, we investigated the regulatory mechanism of the salt stress-tolerance of *AtRH17* OXs using RNA-Seq analysis. AtRH17 is a member of the DEAD-box RHs, which function in RNA metabolism, ribosome biogenesis, and transcriptional and translational regulation [1,2,17]. Previous studies have shown that some DEAD-box RHs play important roles in the response of plants to abiotic stress such as cold, salt, and osmotic stresses as well as in the development of plants [11,12,13,14,15,16,22,23,24,25]. However, abiotic stress-responsive pathways, in which DEAD-box RH genes are involved, have not been well identified and are limited to a few stress-responsive DEAD-box RH genes such as *AtRH38*/*LOS4*, *AtRH42*/*RCF1*, *STRS1*, and *STRS2* [13,14,15,16]. In addition, the identified abiotic stress-responsive pathways mediated by DEAD-box RH genes are restricted to the well-known ABA-dependent and/or ABA-independent pathways [13,14,15,16].

RNA-Seq, a high-throughput and next-generation sequencing technology, has allowed for rapid analysis of large genomic datasets and quantification of transcriptomes [26]. In addition, RNA-Seq analysis can be used to identify and quantify transcripts without prior information of genes and can provide information regarding alternative splicing and sequence variations in identified genes [26,27]. Global gene expression patterns have been determined using RNA-Seq analysis of samples at different developmental stages, in response to different stimuli or in different genotypes [28,29]. RNA-Seq analysis of *AtRH17* OXs revealed that 286 genes were upregulated and 111 genes were downregulated in *AtRH17* OXs relative to WT (Figure 1). The upregulated and downregulated genes were classified into six and four individual groups by hierarchy analysis, respectively (Figure 4). GO annotation enrichment analysis showed that *AtRH17* might regulate genes that are involved in various functions such as signaling, secretion, detoxification, and transcription, depending on enriched GO terms such as response to toxic substances, toxin catabolic process, glutathione metabolic process, oxidation–reduction process, transcription factor activity, RNA polymerase II complex, and extracellular region (Supplementary Figures S1 and S2). KEGG pathway analysis showed that *AtRH17* might be involved in metabolic pathways, biosynthesis of secondary metabolites, plant hormone signal transduction, and MAPK signaling pathway via the regulation of downstream genes functioning in these pathways (Figure 2 and Figure 3).

Previously, we reported that the expression levels of the well-known ABA-dependent and ABA-independent salt stress-responsive genes such as *RD29A*, *RAB18*, *RD29B*, *RD22*, *COR47*, *DREB2A*, and *DREB2B* were not higher in *AtRH17* OXs than in WT under salt stress condition [17]. Using RNA-Seq analysis, we confirmed our previous results that the expression levels of these seven genes were not significantly increased in *AtRH17* OXs (data not shown), indicating that the salt-tolerance in *AtRH17* OXs is mediated through pathway(s) other than the well-known ABA-dependent and/or ABA-independent stress-responsive pathways.

Analysis of the upregulated genes in *AtRH17* OXs using GO annotation and Genevestigator revealed that nine genes, namely, *LEA4-5*, *GSTF6*, *DIN2*/*BGLU30*, *TSPO*, *GSTF7*, *LEA18*, *HAI1*, *ABR*, and *LTI30*, are salt stress-responsive genes and might be involved in the salt-tolerance in *AtRH17* OXs (Table 5). These nine genes are tightly inter-connected (Figure 8). Among these, *LEA4-5*, *TSPO*, and *ABR* were selected for quantitative RT-PCR analysis because their expression increased under all abiotic stress conditions examined in Genevestigator analysis and they showed high expression levels in *AtRH17* OXs (Figure 6 and Table 5). Three genes showed higher expression levels in *AtRH17* OXs than in WT under salt stress conditions (Figure 7). LEA4-5 is a member of LEA proteins. The C-terminal region of LEA4-5 is responsible for its antioxidant activity and in the scavenging of metal ions under stress conditions, whereas the N-terminal can function as a chaperone in the folding of enzyme proteins and in preventing their unfolding that results in protection of the enzyme activity [19,30]. TSPO, belonging to the Trp-rich sensory protein/peripheral-type benzodiazepine receptor group protein, interacts with and regulates PIP2;7, a plasma membrane aquaporin, in the endoplasmic reticulum and Golgi membrane during abiotic stress conditions [20,21]. The interaction between TSPO and PIP2;7 triggers the reduction of PIP2;7 channels present in the plasma membrane and reduces the water transport activity [20,21]. ABR is a Ser/Thr kinase and interacts with phospholipase D-derived phosphatidic acid (PA) [31,32]. ABR increases the ABR-dependent phosphorylation of PIN2, which activates auxin efflux, alters auxin accumulation, and promotes root growth under salt stress conditions [31,32]. T-DNA insertional mutants of *ABR* are hypersensitive to salt stress in primary root elongation and do not respond to PA [31,32]. These results suggest that the upregulated *LEA4-5*, *TSPO*, and *ABR* confer salt stress-tolerance in *AtRH17* OXs.

Taken together, our results demonstrate that *AtRH17* OXs exhibit salt stress-tolerance through a novel salt stress-responsive pathway involving *LEA4-5*, *GSTF6*, *DIN2*/*BGLU30*, *TSPO*, *GSTF7*, *LEA18*, *HAI1*, *ABR*, and *LTI30*, other than the well-known ABA-dependent and ABA-independent pathways.

## 4. Materials and Methods 

### 4.1. Plant Materials and Growth Conditions

All plant materials used in this study were of the *Arabidopsis thaliana* accession Col-0 background. Sterilized seeds were incubated in the dark for 2–3 days at 4 °C and then germinated, grown on half-strength Murashige and Skoog (MS) agar plates, supplemented with 1.5% (w/v) sucrose and B5 vitamin. Seedlings were grown under short-day (SD) conditions (8 h light:16 h dark photoperiod) at 22 °C. 

### 4.2. Plant Stress Treatment for Quantitative RT-PCR

For the analysis of salt stress-responsive gene expression under salt stress conditions, 10-day-old WT and *AtRH17* OX seedlings grown under SD conditions were placed on filter papers soaked in MS solution containing 150 mM NaCl. After 0, 1, 2, 4, and 8 h, the seedlings were harvested; seedlings harvested at 0 h were used as the control.

### 4.3. RNA Isolation and First Strand cDNA Synthesis

Total RNA was extracted using an RNAqueous RNA Isolation Kit (Invitrogen, Carlsbad, CA, USA), supplemented with Plant RNA Isolation Aid (Invitrogen, Carlsbad, CA, USA), according to the manufacturers’ instructions. Two micrograms of total RNA was reverse-transcribed in a total volume of 25 μL containing 0.5 μg of oligo dT primer, 0.5 mM dNTPs, and 200 units of *Moloney Murine Leukemia Virus* (*M-MLV*) reverse transcriptase (Promega, Madison, WI, USA).

### 4.4. Quantitative RT-PCR

Quantitative RT-PCR analysis was performed using 2× POWER SYBR Green PCR Master mix (Applied Biosystems, Foster, CA, USA) with diluted cDNA as the template. The C_t_ (cycle at the threshold) value was set to be constant throughout the study and corresponded to the log-linear range of PCR amplification. The normalized amount of target reflected the relative amount of target transcripts with respect to that of the endogenous reference gene, *GAPc*. The amplification conditions included an initial denaturation at 95 °C for 10 min, followed by repeated cycles at 95 °C for 30 s, 60 °C for 30 s, and 72 °C for 30 s. The primers used are listed in Supplementary Table S1.

### 4.5. Library Preparation and RNA-Sequencing

The RNA quality was assessed with Agilent 2100 bioanalyzer using the RNA 6000 Nano Chip (Agilent Technologies, Santa Clara, CA, USA), and RNA quantification was performed using a ND-2000 Spectrophotometer (Thermo Scientific, Waltham, MA, USA). For the control and test RNAs, a library was constructed using QuantSeq 3′ mRNA-Seq Library Prep Kit (Lexogen, Inc., Vienna, Austria), according to the manufacturer’s instructions. In brief, 500 ng total RNA was hybridized to an oligo dT primer containing an Illumina-compatible sequence at its 5′-end and reverse transcription was performed. After degradation of the RNA template, second strand synthesis was initiated using a random primer containing an Illumina-compatible linker sequence at its 5′-end. The double-stranded library was purified of the reaction components by magnetic separation. The library was amplified to add the complete adapter sequences required for cluster generation, and the finished library was purified from the PCR components. High-throughput single-end 75 sequencing was performed using NextSeq 500.

### 4.6. RNA-Seq Data Analysis

QuantSeq 3′ mRNA-Seq reads were aligned using Bowtie2 [33]. Bowtie2 indices were either generated from the genome assembly sequence or representative transcript sequences for aligning to the genome and transcriptome. The alignment file was used for assembling the transcripts, estimating their abundance, and detecting the differential expression of genes. DEGs were determined based on the counts from unique and multiple alignments using coverage in Bedtools [34]. The Read Count data were processed based on the quantile normalization method employing EdgeR within R [35] using Bioconductor [36]. GO annotation enrichment was performed using DAVID [37] with default parameters. KEGG pathway analysis was conducted using KEGG Mapper [38] Gene clustering was performed using MeV ver. 4.9.0 [39]. Protein network analysis was performed using String Apps of Cytoscape ver. 3.7.2 [40].

### 4.7. Statistical Analysis

Statistical analysis was performed using one-way analysis of variance and Dunnett’s post-hoc test, as implemented in IBM SPSS v.23 (IBM Corp., Armonk, NY, USA).

## Figures and Tables

**Table 1:** Summary of mapping transcriptome reads to reference sequence.

Sample	Total Read	Processed Read	Mapped Read	Mapping Rate
WT-1	31,584,235	30,119,902	29,518,240	98.01%
WT-2	32,020,154	30,339,538	29,616,323	97.62%
*AtRH17* OX-1	32,659,954	30,992,415	30,299,369	97.76%
*AtRH17* OX-2	28,802,597	27,060,251	26,375,818	97.47%

**Table 2:** Biological process categories of Gene Ontology (GO) annotation of upregulated genes in *AtRH17* OXs.

GO Term	Description	Number in Input List	*p*-Value
GO:0055144	oxidation–reduction process	34	0.00000
GO:0050832	defense response to fungus	14	0.00095
GO:0006952	defense response	14	0.01700
GO:0006979	response to oxidative stress	12	0.00020
GO:0009651	response to salt stress	11	0.00600
GO:0009636	response to toxic substance	10	0.00000
GO:0009414	response to water deprivation	10	0.00230
GO:0009407	toxin catabolic process	9	0.00000
GO:0006749	glutathione metabolic process	9	0.00000
GO:0006869	lipid transport	9	0.00015
GO:0009611	response to wounding	9	0.00100
GO:0009617	response to bacterium	8	0.00008
GO:0009813	flavonoid biosynthetic process	8	0.00071
GO:0042742	defense response to bacterium	8	0.02300
GO:0051707	response to other organism	7	0.00002
GO:0006357	regulation of transcription from RNA polymerase II promoter	7	0.02200
GO:0071456	cellular response to hypoxia	6	0.00001
GO:0010150	leaf senescence	6	0.00250
GO:0080167	response to karrikin	6	0.01000
GO:0045454	cell redox homeostasis	6	0.02100
GO:0042744	hydrogen peroxide catabolic process	5	0.01300
GO:0009826	unidimensional cell growth	5	0.02900
GO:0002213	defense response to insect	4	0.00063
GO:0009682	induced systemic resistance	4	0.00075
GO:0006032	chitin catabolic process	4	0.00200
GO:0006040	amino sugar metabolic process	3	0.00990
GO:0000272	polysaccharide catabolic process	3	0.01700
GO:0071732	cellular response to nitric oxide	3	0.01700
GO:0042343	indole glucosinolate metabolic process	3	0.01900
GO:0016998	cell wall macromolecule catabolic process	3	0.02800
GO:0055072	iron ion homeostasis	3	0.04200
GO:0045168	cell-cell signaling involved in cell fate commitment	3	0.04200
GO:0046256	2,4,6-trinitrotoluene catabolic process	2	0.02000
GO:0006801	superoxide metabolic process	2	0.02000
GO:0009790	embryo development	2	0.03000
GO:0071457	cellular response to ozone	2	0.03000

**Table 3:** Biological process categories of GO annotation of downregulated genes in *AtRH17* OXs.

GO Term	Description	Number in Input List	*p*-Value
GO:0055114	oxidation-reduction process	11	0.0330
GO:0006952	defense response	7	0.0430
GO:0009664	plant-type cell wall organization	6	0.0000
GO:0006979	response to oxidative stress	5	0.0270
GO:0042744	hydrogen peroxide catabolic process	4	0.0051
GO:00009741	response to brassinosteroid	3	0.0077
GO:0009739	response to gibberellin	3	0.0570
GO:0009826	unidimensional cell growth	3	0.0730
GO:0009723	response to ethylene	3	0.0840
GO:0009641	shade avoidance	2	0.0570
GO:0010017	red or far-red light signaling pathway	2	0.0930

**Table 4:** List of 19 genes upregulated in *AtRH17* OXs, enriched in “response to salt” (GO:000965) and “response to water deprivation” (GO:0009414).

Locus ID	Gene Symbol	Fold Change	*p*-Value	Stress Responses
At1g02930	GSTF6	10.377	0.000	salt, water deprivation
At5g10230	ANNAT7	3.366	0.009	salt, water deprivation
At1g10370	GSTU17	9.761	0.004	salt
At3g60140	DIN2/BGLU30	8.008	0.008	salt
At2g47770	TSPO	7.078	0.032	salt
At1g02920	GSTF7	6.927	0.002	salt
At2g47460	MYB12	4.418	0.006	salt
At4g14630	GLP9	4.116	0.001	salt
At5g24090	CHIA	3.627	0.007	salt
At2g38390	PER23	3.387	0.017	salt
At1g08830	CSD1	3.087	0.013	salt
At5g06760	LEA4-5	10.426	0.009	water deprivation
At5g59220	HAI1	6.705	0.001	water deprivation
At2g35300	LEA18	6.182	0.010	water deprivation
At3g02480	ABR	5.526	0.003	water deprivation
At1g69600	ZFHD1	4.178	0.014	water deprivation
At3g45140	LOX2	4.061	0.027	water deprivation
At3g50970	LTI30	3.502	0.018	water deprivation
At5g07690	MYB29	3.325	0.022	water deprivation

**Table 5:** List of nine stress-responsive genes selected from Genevestigator analysis.

Locus ID	Gene Symbol	Fold Change	*p*-Value	Description
At5g06760	LEA4-5	10.426	0.009	Member of the Late Embryogenesis Abundant (LEA) protein
At1g02930	GSTF6	10.377	0.000	Glutathione transferase belonging to the phi class of GSTs
At3g60140	DIN2/BGLU30	8.008	0.008	Glycoside hydrolase superfamily protein
At2g47770	TSPO	7.078	0.032	Membrane-bound protein, TSPO-related protein
At1g02920	GSTF7	6.927	0.002	Glutathione transferase belonging to the phi class of GSTs
At2g35300	LEA18	6.182	0.010	Member of the Late Embryogenesis Abundant (LEA) protein
At5g59220	HAI1	6.705	0.001	Member of the PP2C family (Clade A protein phosphatases type 2C)
At3g02480	ABR	5.526	0.003	Late embryogenesis abundant protein (LEA) family protein
At3g50970	LTI30	3.502	0.018	Dehydrin protein family

## Supplementary Materials

Supplementary materials can be found at https://www.mdpi.com/1422-0067/21/5/1595/s1.

## Abbreviations

ABA	Abscisic acid
DAG	Days after germination
DEG	Differentially expressed gene
GO	Gene Ontology
KEGG	Kyoto Encyclopedia of Genes and Genomes
LD	Long-day
MS	Murashige and Skoog
OX	Overexpressing transgenic plant
RH	RNA helicase
SD	Short-day
