# Supplementary material for: Investigation of a Novel Salt Stress-Responsive Pathway Mediated by Arabidopsis DEAD-Box RNA Helicase Gene AtRH17 Using RNA-Seq Analysis
Source: Int J Mol Sci. 2020 Feb 26;21(5):1595. doi: 10.3390/ijms21051595 (PMC7084250; doi:10.3390/ijms21051595)
Supplement: Supplementary file 1 [file ijms-21-01595-s001.pdf]

Supplementary data

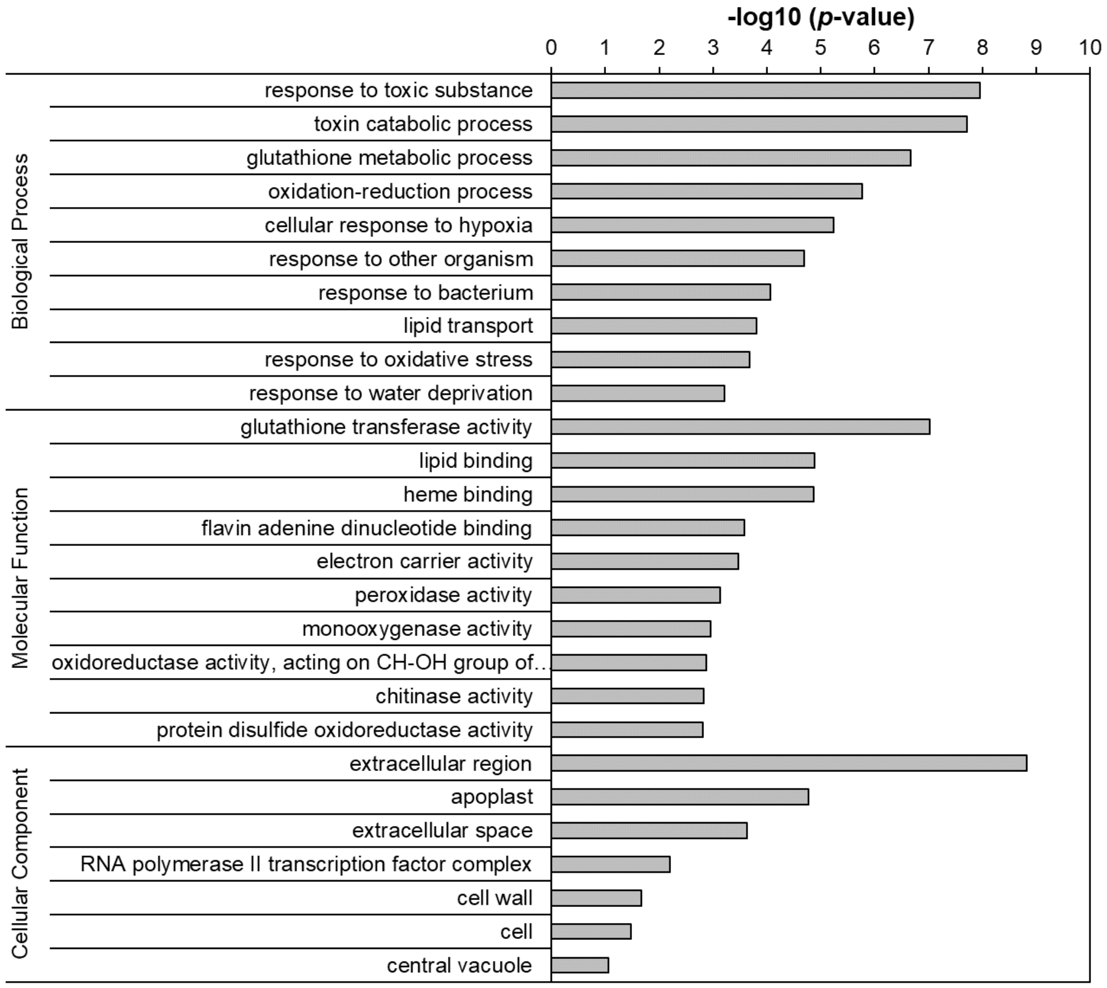

Figure S1. Functional classification of GO terms of up-regulated genes in *AtRH17* OXs.

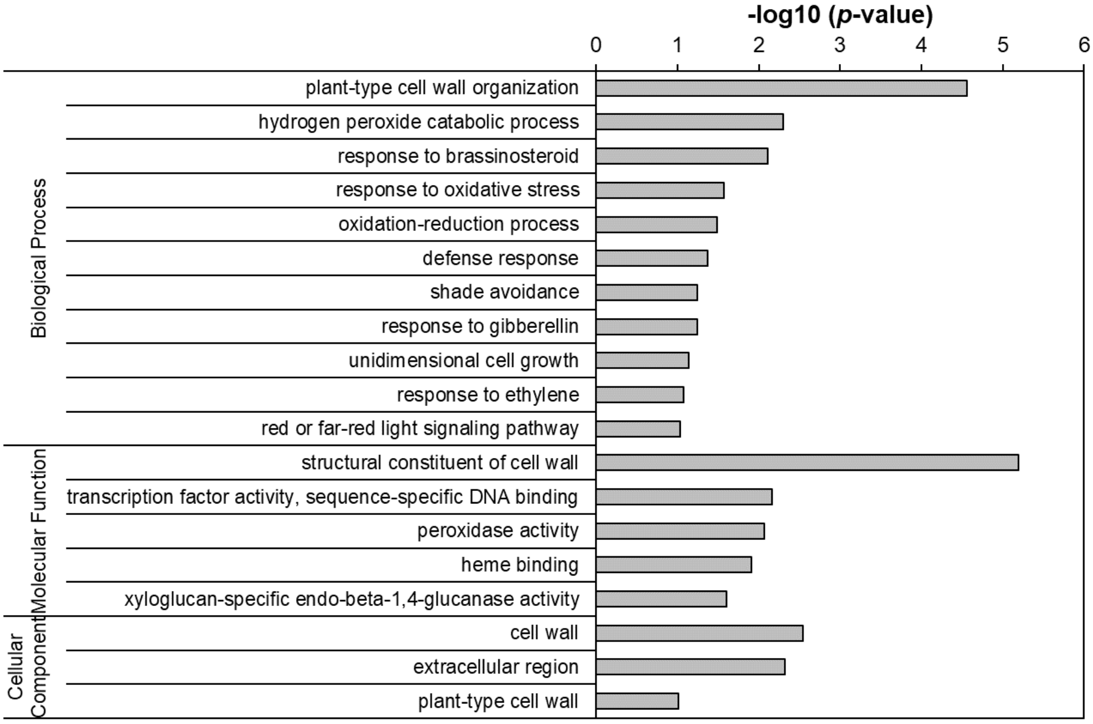

Figure S2. Functional classification of GO terms of down-regulated genes in *AtRH17* OXs.

Table S1. List of primers used for quantitative RT-PCR

Gene	Forward	Reverse
AtRH17	5´-TTCCCATACCCGGCAATATG-3´	5´-TCGGCGTAAGTAAGGGATTC-3´
GAPc	5´-GTGTCCCAACCGTTGATGTC-3´	5´-TCCCTTGAGTTTGCCTTCGG-3´
TSPO	5´-AAGCGTGTGAGGAGGCGTCG-3´	5´-TACGTGTCTCGTCGTAGATC-3´
LEA4-5	5´-GTGACCGACCCGATTGGAAG-3´	5´-CCCCGCCGGTTCCGTACCCG-3´
ABR	5´-AGGAGAAGGCCGGTGGAATG-3´	5´-GTCCTTGACGACATCAGAAG-3´
LEA18	5´-ATCTTCCCGGTCACTCCACC-3´	5´-GCGGCTAATCTTAATATAGC-3´
DIN2	5´-ATCTTCCCGGTCATCCCACC-3´	5´-GCGGCTAATCTTAATATAGC-3´
PIL1	5´-CCTACGATGTTGCCAATGGG-3´	5´-GAATTGCGGTATTGATGTTG-3´
FRF4	5´-CCTCGCGAGGCTCGCCATAC-3´	5´-CATCTAACTGTTCCTTGTAC-3´
MYB108	5´-GAGGATTCAGTCAGCCTCGG-3´	5´-CGTGCCGGCTGATCCTGTGG-3´
NAC019	5´-GCATAATAACTCGGTACCGG-3´	5´-CGTGACTGCTCTCGACTTCC-3´
RD29A	5´-CCTGAAGTGATCGATGCACC-3´	5´-CAGTGGGTTTGGTGTAATCG-3´
